# Mechanical Properties Analysis of Flexible Memristors for Neuromorphic Computing

**DOI:** 10.1007/s40820-025-01825-x

**Published:** 2025-07-17

**Authors:** Zhenqian Zhu, Jiheng Shui, Tianyu Wang, Jialin Meng

**Affiliations:** 1 School of Integrated Circuits, Shandong University, Jinan, 250100 People’s Republic of China; 2 Suzhou Research Institute of Shandong University, Suzhou, 215123 People’s Republic of China; 3 State Key Laboratory of Crystal Materials, Shandong University, Jinan, 250100 People’s Republic of China; 4 National Integrated Circuit Innovation Center, Shanghai, 201203 People’s Republic of China

**Keywords:** Flexible memristor, Neuromorphic computing, Mechanical property, Wearable electronics

## Abstract

The advancement of flexible memristors has significantly promoted the development of wearable electronic for emerging neuromorphic computing applications. Inspired by in-memory computing architecture of human brain, flexible memristors exhibit great application potential in emulating artificial synapses for high-efficiency and low power consumption neuromorphic computing. This paper provides comprehensive overview of flexible memristors from perspectives of development history, material system, device structure, mechanical deformation method, device performance analysis, stress simulation during deformation, and neuromorphic computing applications. The recent advances in flexible electronics are summarized, including single device, device array and integration. The challenges and future perspectives of flexible memristor for neuromorphic computing are discussed deeply, paving the way for constructing wearable smart electronics and applications in large-scale neuromorphic computing and high-order intelligent robotics.

## Highlights

- This review systematically summarizes materials system, development history, device structure, stress simulation and applications of flexible memristors.
- This review highlights the critical influence of mechanical properties on flexible memristors, with particular emphasis on deformation parameters and finite element simulation.
- The applications of future memristors in neuromorphic computing are deeply discussed for next-generation wearable electronics

## Graphical abstract

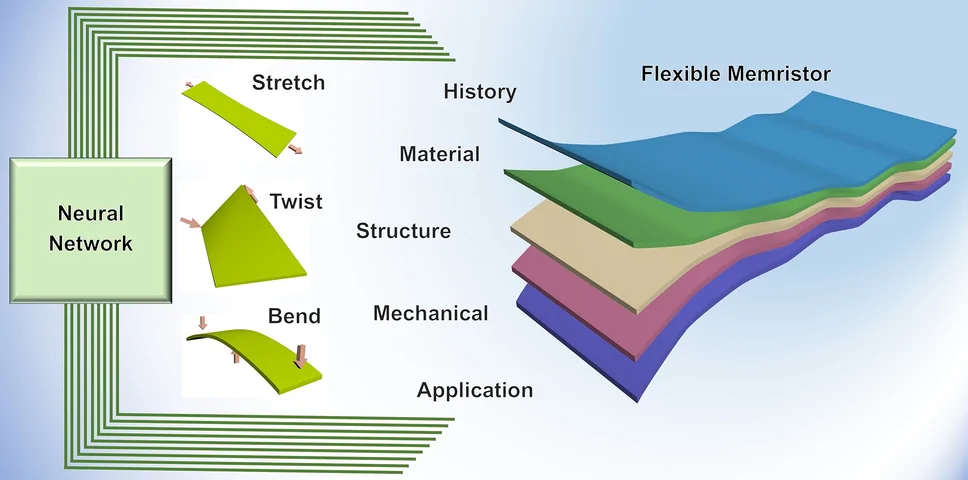

## Introduction

**Fig. 1:**
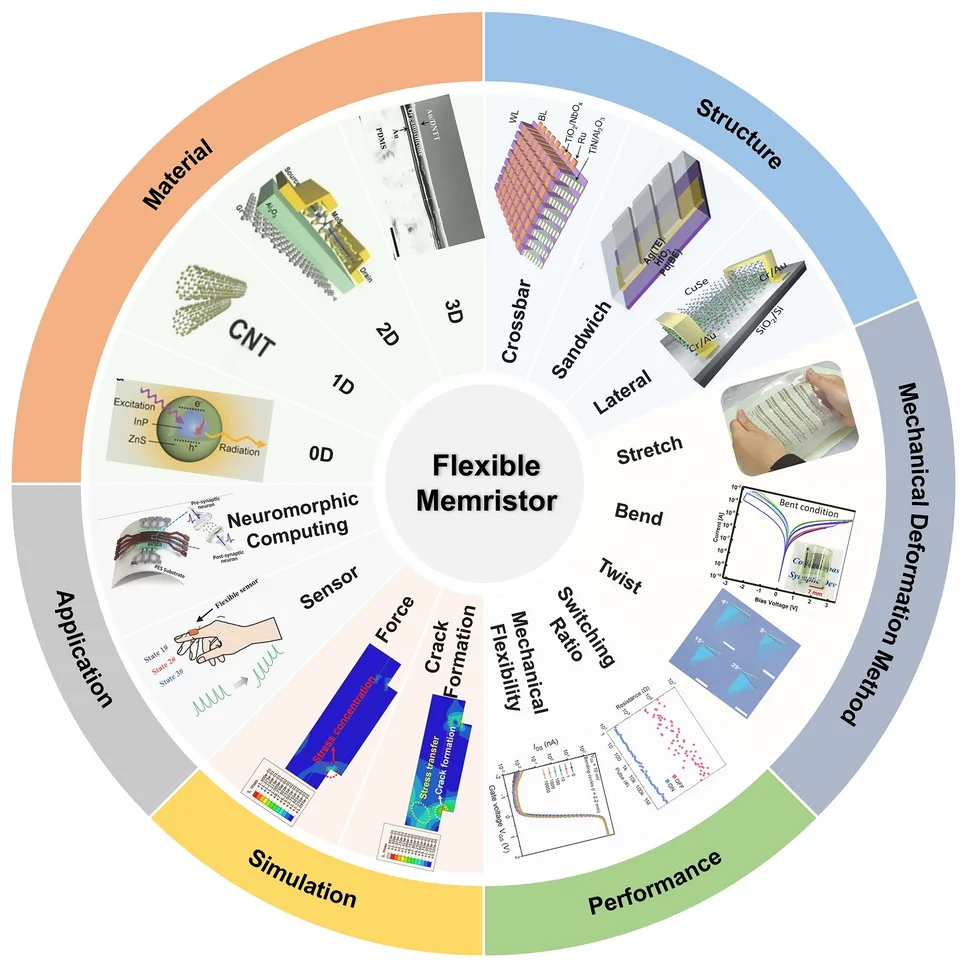
Summary of this review, including materials, structure, mechanical deformation method, performance, stress simulation and application of flexible memristors. The material type includes 3D structure [1], Copyright (2019) Wiley–VCH. 2D structure [2], Copyright (2017) Wiley–VCH. 1D structure [3], Copyright (2024) Wiley–VCH. and 0D structure [4], Copyright (2020) Wiley–VCH. The common structure of the flexible memristor, including crossbar, sandwich and lateral structure [5–7]. Copyright (2024) American Chemical Society. Copyright (2023) American Chemical Society. Copyright (2022) Wiley–VCH. Mechanical deformation method of flexible electronics, including stretching [8], Copyright (2022) The Authors. bending [9], Copyright (2018) Wiley–VCH, and twisting [10]. Copyright (2024) Wiley–VCH. The key performance of flexible memristor, including switching ratio [11], Copyright (2024) The Authors. Published by American Chemical Society, and mechanical flexibility [12], Copyright (2025) Wiley–VCH. The simulation of flexible electronics for force concentration analysis and crack formation process [13]. Copyright (2024) The Authors. Flexible applications to neuromorphic computing and sensors [14, 15]. Copyright (2022) Wiley–VCH. Copyright (2024) Wiley–VCH

With the rapid development of the Internet of Things (IoT) and Artificial Intelligence (AI) technology, traditional silicon-based transistors are gradually facing physical limits in terms of flexibility, integration density and energy consumption. As an emerging device integrating flexible electronic technology and memory characteristics, flexible memristor exhibits great potential in information storage and neuromorphic computing (Fig. 1). The memristor provides a novel physical hardware for breaking through the bottleneck of traditional von Neumann architecture. With in-memory computing architecture [16, 17], non-volatile switching characteristics and synaptic behavior [15, 18–23], memristor promotes the development of neuromorphic computing [24–33]. The introduction of flexible substrates and stretchable structures enables memristor to adapt to bending [24, 34–39], twisting [40], stretching operations and so on, thus showing unique advantages in flexible sensor [27, 41–43], flexible memory [44, 45], implantable electronics and textile electronics [46–49]. Through innovative design of oxide materials, organic materials [22, 30, 50–52] and two-dimensional materials [14, 24, 37, 45, 53–58], flexible memristors have made remarkable breakthroughs in key properties. However, large-scale manufacturing and implementation of neuromorphic computing system still face challenges [59]. Recent advances of flexible memristor have shown significant potential in neuromorphic computing applications, leveraging non-volatile, high speed and low-power consumption characteristics, as summarized in Table 1. In IoT and wearable systems, flexible memristors facilitate personalized signal processing with high classification accuracy, biocompatibility and adaptability. For edge computing and medical diagnostics, flexible memristors offer ultra-low power solutions with real-time processing capabilities. Additionally, fault-tolerant architectures enhance reliability in large-scale crossbar arrays, paving the way for high-density integration. However, key challenges such as material stability, multi-level storage and fabrication scalability must be addressed to constructing energy-efficient flexible intelligent systems.

**Table 1:** State-of-the-art memristor applications

Refs	Application domain	Test case	Performance advantage vs. baseline	Limitations
[60]	Non-von Neumann computing (PIM)	MAGIC NOR operations in 1S1R arrays (4 × 4 to 512 × 512) with VO₂ selector & HfOₓ RRAM	21.9 × lower power (NOR(0,0)) & 24.4 × lower power (NOR(0,1)/NOR(1,0)/NOR(1,1)) vs. 1R arrays 4.5 × higher readout margin vs. 1R arrays Eliminates sneak-path currents	Switching delay increases with array size Requires selector integration (fabrication complexity)
[61]	In-Memory Computing	SPICE simulations with 45 nm CMOS	Eliminates sneak-path currents Supports all-to-all connectivity Non-volatile configuration	RC delay in large arrays Variability challenges
[62]	Integration with IoT and wearable technology	Creeping wave analysis around human torso using cross-slot antennas	Exponential decay model for personalized EM wave propagation 90% classification accuracy for cloth size prediction	Sensitivity to body dimensions/dielectric properties Hand proximity degrades signal
[63]	Neuromorphic Hardware Design	DAC/ADC optimization via state/resolution analysis	Reduces DAC bits by 83% (48 → 8 states) Cuts ADC bits from 13 to 6 for 10 μA resolution	SCL (Same Current Level) jumps to 29.1% at 50 μA resolution for Wine dataset
[64]	Neuromorphic Computing	64 × 64 memristor crossbar with adaptive threshold calibration	Accuracy improvement from 49 to 80% in character recognition Mismatch compensation via dynamic Vth adjustment	Requires per-neuron post-fabrication characterization
[65]	In-Memory Computing	Voltage-divider based IMP gate (P-Q anti-serial connection)	0.5 V read voltage achieves 0.37–0.68 V output range No switching required (energy < 1 nJ/op)	Asymmetry causes 23% output deviation (ideal 0.5 V → factual 0.37–0.68 V)
[66]	Edge Computing & Mobile Devices	11 ML datasets	4% higher accuracy and 12.6% lower energy vs. SOTA energy-efficient inference (10 fJ/memristor)	Trade-off between multivariate node accuracy and circuit complexity
[67]	Compute-in-Memory (CIM) Systems	10 ns write-speed validation	10ns ultrafast write speed; > 1000 × scalable resistance (RA) for analog computing	Requires controlling of film thickness for optimal resistance
[68]	Multimodal Neuromorphic Computing	Video recognition tasks (image + sound)	6 TOPS/mm^2^ area efficiency 98% classification accuracy 1.7pJ/operation energy	Limited to 3D RRAM array implementation Requires specialized fabrication process
[69]	Edge Medical Diagnostics	Blood cell classification (RBC/WBC/PLT)	1.547 TOPS/W energy efficiency 1142 frames/sec recognition rate 90% accuracy after 6000 h	Low on/off ratio
[70]	Fault-Tolerant Memristor Crossbar Systems	Defect mapping in 128 × 128 crossbars	99.9992% uptime ratio Average 15.24 years functional correctness Simultaneous row/column permutation	0.2% fabrication defect rate impacts yield Limited by endurance cycles
[71]	GNN Acceleration	Cora (2,708 nodes)	35,500 × energy improvement vs GPU 0.9% accuracy loss Single-step neighborhood retrieval	Conductance variation tolerance

**Fig. 2:**
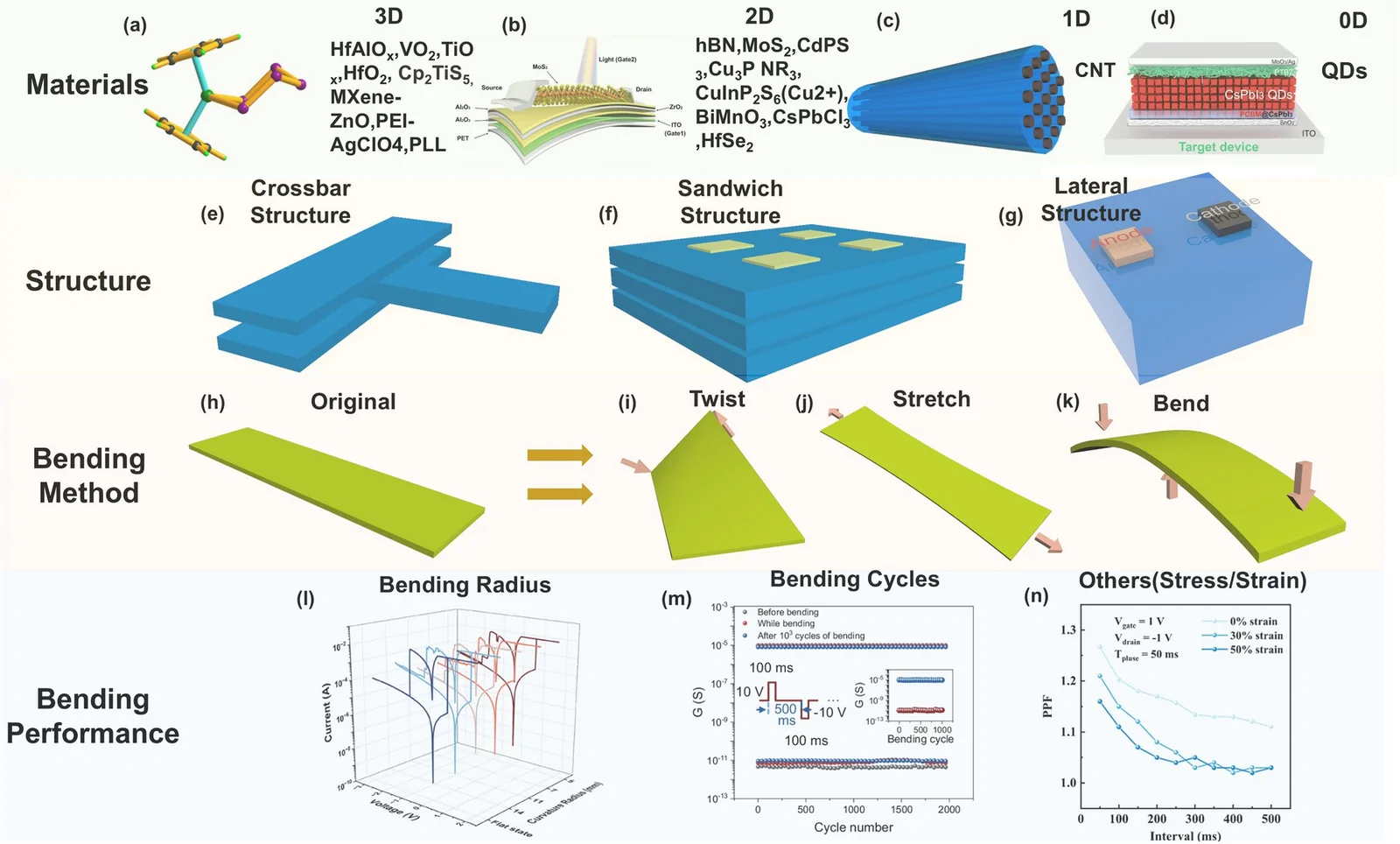
Materials system, device structure, bend method and performance of devices. Materials are classified into **a** three dimensions (3D), **b** two dimensions (2D), **c** one dimension (1D) and **d** zero dimension (0D) [72–75]. Copyright (2025) American Chemical Society. Copyright (2020) Wiley–VCH. Copyright (2015) American Chemical Society. Copyright (2021) The Authors. The typical structure of memristor includes **e** crossbar structure, **f** sandwich structure and **g** lateral structure. **h** Memristor under initial state. The bending methods of flexible memristor, including **i** twisting, **j** stretching, and **k** bending operations. The key performance of flexible memristor under different **l** bending radius, **m** bending cycles, and **n** stress/strain operations [76–78]. Copyright (2023) Wiley–VCH. Copyright (2024) The Authors. Copyright (2022) American Chemical Society

To improve the performance of flexible memristor, materials, structure, bending method and flexible performance should be clarified. As shown in Fig. 2, the material system can be divided into four types, including 3D bulk phase materials (such as HfAlO [47, 79], VO₂ [25, 80, 81], TiO₂ [5], organic material [9, 82, 83]), 2D layered materials (hBN [55, 84], MoS₂ [38, 43, 85–87], MXene [88]), 1D nanowires/tubes (carbon nanotubes CNT [3, 89]), and 0D quantum dots (CsPbCl₃ [90], core–shell QDs [91]). Among those, low-dimensional materials provide unique advantages for uniform film formation and stress release on flexible substrates due to high specific surface area and mechanical flexibility [20, 37, 38, 92]. The 0D quantum dots optimize the interface charge transport properties through the quantum confinement effect [4, 90, 91, 93]. The structure of flexible memristor could be divided into three parts, including crossbar array, traditional sandwich and lateral structure [38, 94, 95]. The sandwich structure enhances the interface stability through the symmetrical design of the electrode/dielectric layer/electrode [96, 97]. It is interesting that some textile memristors were proposed with above structure for wearable applications [42, 48, 98]. An in-depth analysis of the performance characteristics of flexible memristors was presented with three primary forms of mechanical deformation, including bending, stretching and twisting states. Key parameters associated with mechanical deformation were measured to analyze stress distribution and mechanical properties of the device. The effects of mechanical deformation behavior on properties of the device were important for identifying failure mechanisms [24, 37, 38, 92, 99–103]. Based on the failure mechanisms, various strategies for enhancing the mechanical and electrical properties of flexible memristors were explored [55, 95, 104–108]. In addition, mechanical simulations methods were developed to provide guidelines for improving the flexible tolerance of memristors, such as finite element simulation [13, 43].

This review provides a comprehensive overview of flexible memristor for neuromorphic computing and smart wearable electronics. The development history, material system, and device structure design are systematically summarized for prepare high-performance flexible memristor. Mechanical deformation method, device performance analysis, and stress simulation during deformation are discussed for improving reliability of flexible memristor. Finally, the applications of the flexible memristors in neuromorphic computing are discussed deeply [22, 27, 31, 42, 46, 49, 50, 79, 81, 87, 88, 91, 109–115], addressing current challenges and proposing potential directions for future development [26, 37, 101, 105, 116–121].

## History of Flexible Memristor Development

**Fig. 3:**
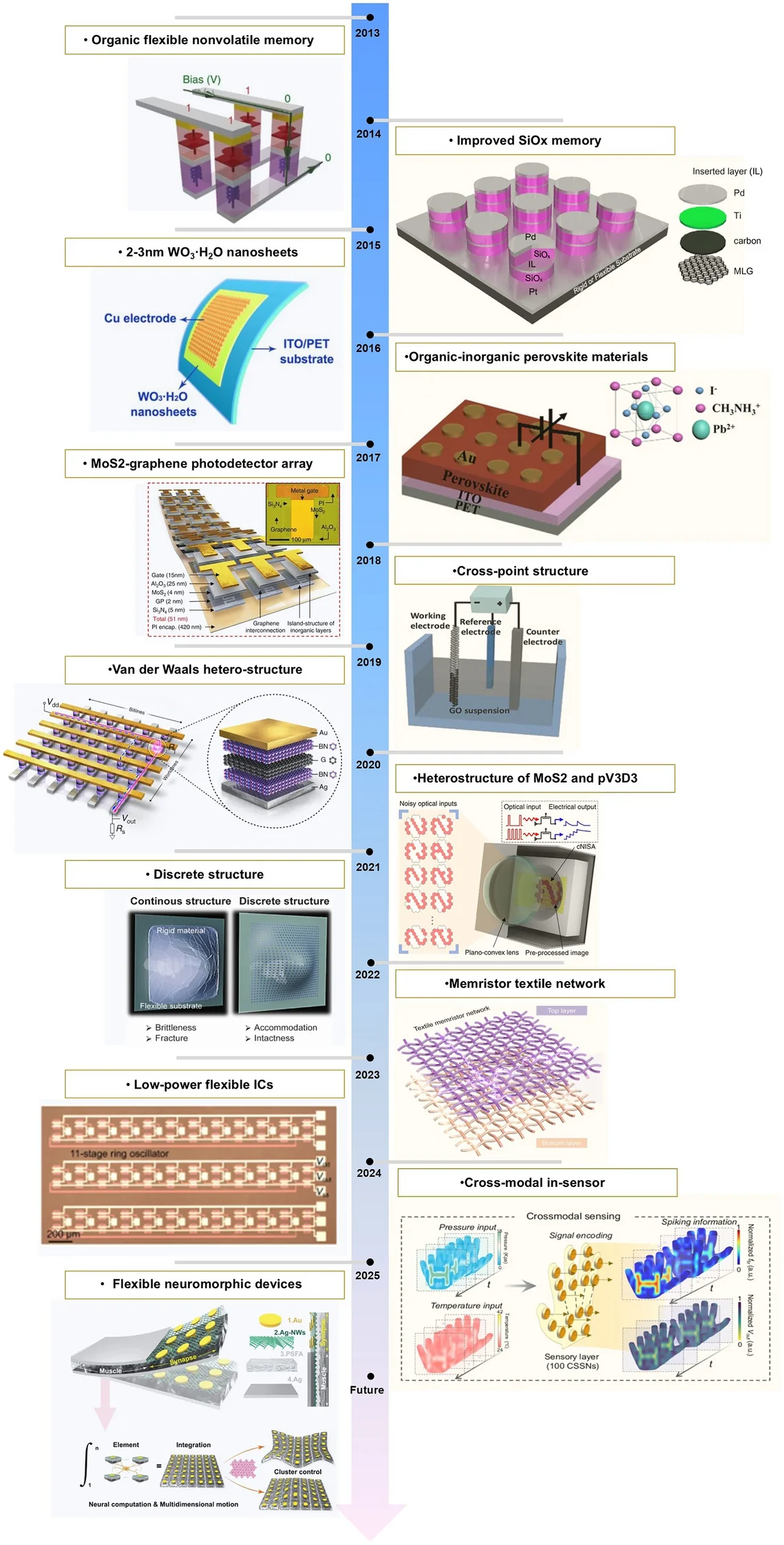
Development of flexible memristors from 2013 to 2025. Organic flexible memory [122]. (Copyright (2013) The Authors), SiO_x_ memory [123] (Copyright (2014) American Chemical Society), WO_3_·H_2_O nanosheets [19] (Copyright (2015) American Chemical Society), and perovskite materials are developed for flexible memristor [124]. (Copyright (2016) American Chemical Society). MoS_2_-graphene photodetector array [43] (Copyright (2017) The Authors), RGO fiber-based device [49] (Copyright (2018) Wiley–VCH), self-selective van der Waals heterostructure device [84] (Copyright (2019) The Authors), heterostructure device of MoS_2_ and pV3D3 [57] (Copyright (2020) The Authors) are proposed for non-volatile memory. Discrete structure [116] (Copyright (2021) Wiley–VCH), reconfigurable textile memristor network [48] (Copyright (2022) The Authors), low-power flexible ICs based on MoS_2_ [85] (Copyright (2023) The Authors), in-sensor reservoir computing system [80] (Copyright (2024) The Authors), and synapse-motor coupler device (SMCD) are developed for emerging in-memory computing applications [125] (Copyright (2025) The Authors)

The flexible memristors entered explosive development period after 2020, which could be traced back to around 2013, as shown in Fig. 3. The integration of flexible electronics and memory technology promotes the development of flexible memristors. From 2013 to 2014, initial efforts focused on adapting traditional materials to flexible substrates, such as SiO_x_ [123]. The initial attempt of flexible organic materials is also carried out, where the thickness was 70 nm [122]. Limited by mechanical brittleness and interface failure issues, few devices can achieve more than 2000 cycles at a bending radius below 5 mm. The rapid growth of the printed electronics industry has accelerated the seeking for materials of flexible memory with film thinner than 3 nm [126]. In 2015, ultra-thin WO_3_·H_2_O nanosheets (2–3 nm) were introduced for the construction of high-performance flexible RRAM [19]. During 2016 to 2017, the field experienced rapid growth with the introduction of two-dimensional materials (e.g., MoS₂) and organic–inorganic hybrid perovskites (e.g., CH_3_NH_3_PbI_3_). These advances demonstrate the implanting of flexible devices and the feasibility of operation under prolonged deformation. Stable performance under the bending radius of 7.5 mm was demonstrated, where switching ratio has no obvious attenuation after 100 bending cycles [43, 124]. Park et al. proposed a flexible biological memristor device based on lignin, providing a new natural material approach for flexible memristors [127]. Various flexible substrates were introduced to fabricate flexible memristor, such as polyethylene terephthalate (PET) and polyimide (PI) [128]. In 2018, an artificial synapse based on reduced oxidized graphene (RGO) was proposed [49], which was one step closer to realizing flexible neuromorphic computing. In 2019, vertical heterostructure self-selective memory based on h-BN with self-selectivity of 10^1^⁰ was introduced [84], effectively solving the problem of sneak current in large-scale arrays. With the development of flexible artificial synaptic memristors, face recognition on damaged images was successfully achieved by flexible pV3D3 memristor [129].

Inspired by human visual system, flexible neuromorphic image sensor array was proposed for photon-triggered synaptic plasticity and preprocessing noisy images, thereby significantly enhancing the efficiency of image recognition [57]. This innovation paved the way for the development of next-generation machine vision applications. In 2021, stretchable memristor with superior deformability was proposed for operation in harsh environments, which maintained stable performance even after sustaining extreme mechanical injuries such as punctures and severe tears [116]. From 2022 to 2024, studies of flexible memristors turned to realize multimodal perception and wearable IC applications [80, 85]. For the first time, Wang et. al proposed reconfigurable textile memristors with heterostructure of MoS_2_ and HfAlO_x_ for realize functions of artificial synapse and neurons, exhibiting great potential in intelligent neuromorphic computing system [48]. In 2025, synaptic-motor coupler device (SMCD) that integrates neural computing and muscle actuation was proposed, effectively bridging perception and action within neuromorphic system [125]. High-performance flexible materials, advanced manufacturing processes, and reconfigurable memristor networks pave the way for designing next-generation flexible neuromorphic computing electronics [130]. This development trajectory highlighted the evolution of flexible memristors from material substitution to structural innovation and ultimately toward system integration, laying the physical foundation for flexible intelligent hardware in the post-Moore era.

## Material of the Flexible Memristor

**Fig. 4:**
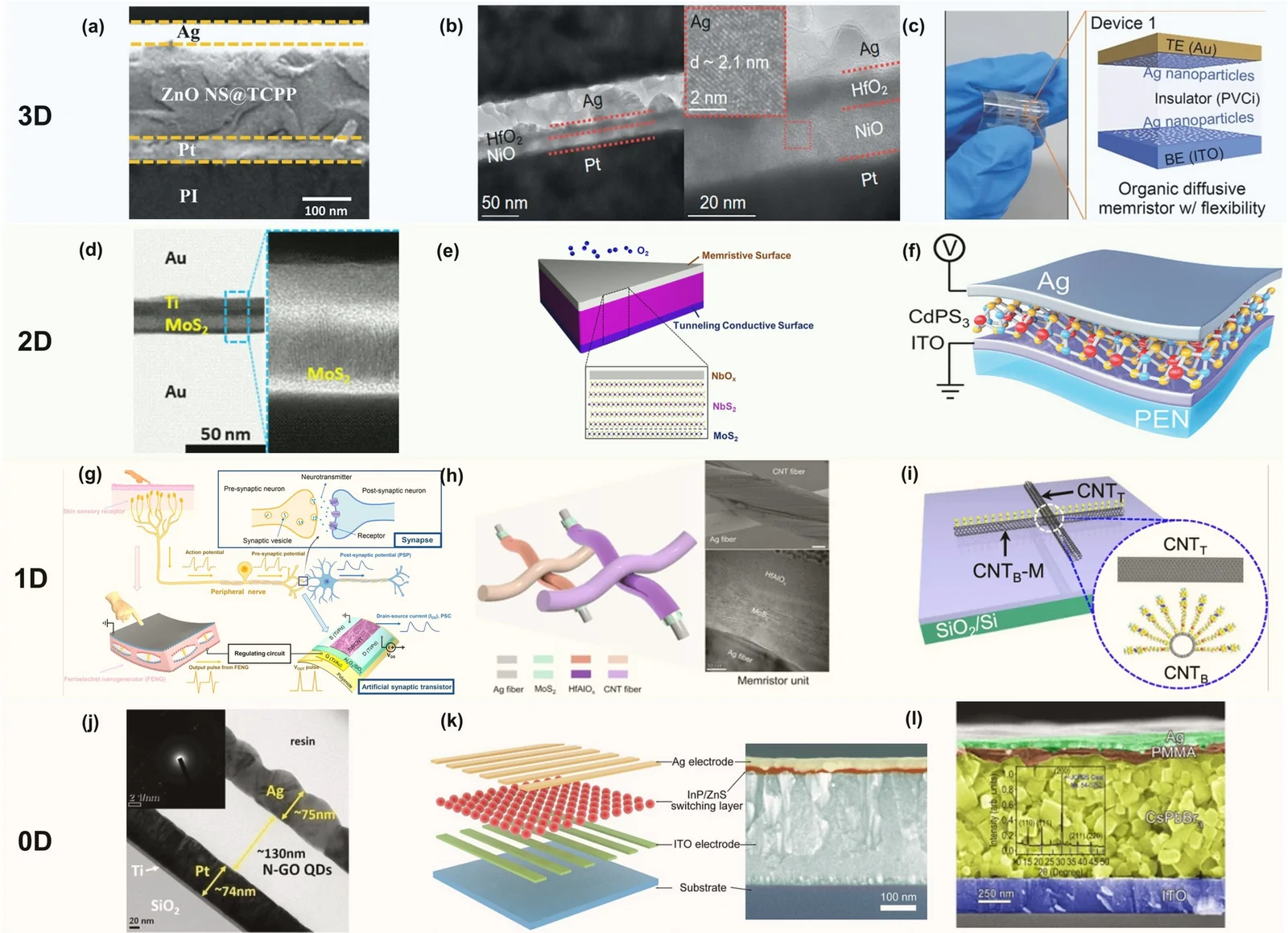
Material type divides to four parts, including 3D, 2D, 1D, and 0D. **a** Cross-section SEM image of the flexible film [20]. Copyright (2022) Wiley–VCH. **b** TEM image of the HfO_2_/NiO films [21]. Copyright (2024) American Chemical Society. **c** Flexible diffusive memristor with organic functional layer [22]. Copyright (2023) Wiley–VCH. **d** TEM cross-sectional image of MoS_2_ [86]. Copyright (2022) American Chemical Society. **e** This diagram of MoS_2_ − NbS_2_ − NbO_x_ heterojunction [131]. Copyright (2020) American Chemical Society. **f** The structure of CdPS_3_-based memristor [14]. Copyright (2022) Wiley–VCH. **g** Artificial skin composed of carbon nanotube [132]. Copyright (2020) American Chemical Society. **h** Structure of the fiber-based memristor, consisting of Ag/MoS_2_/HfAlO_x_/CNT [48]. Copyright (2022) The Authors. **i** CNT_B_ -M/CNT_T_ vdW 1D device [89]. Copyright (2022) The Authors. **j** Cross-sectional STEM image of N-GO QDs [93]. Copyright (2019) Wiley–VCH. **k** Schematic illustration of InP/ZnS QD-based memristor [4]. Copyright (2020) Wiley–VCH. **l** SEM image of CsPbBr_3_ QDs-based LEM [90]. Copyright (2021) The Authors

The 3D materials have made remarkable progress in flexible memristors, especially in neuromorphic computing and wearable electronic systems [133, 134]. The 3D materials can be categorized into organic materials, inorganic materials, and hybrid materials. Hybrid materials-based flexible memristor was fabricated by embedding ZnO nanosheets into PMMA, providing a new approach for low-cost flexible neuromorphic hardware [20], as shown in Fig. 4a. For inorganic materials, the HfO₂/NiO-based memristor exhibited diffusion behavior and reversible switching between volatile and non-volatile modes [21], as shown in Fig. 4b. Figure 4c shows flexible organic memristor with biological synaptic plasticity, including STP and LTP characteristics [22]. The mechanical flexibility and solution-process of organic materials further promote the development of wearable intelligent systems [135]. By fabricating memristor on flexible PET substrate, low-cost flexible electronics exhibit advantages in bending tests [2]. Meanwhile, PI substrate-based flexible memristor was proposed [136], where the performance was comparable to that of devices on rigid substrates.

With advantages of low power consumption and high flexibility, two-dimensional (2D) materials have attracted interests of researchers in flexible electronics. 2D materials are predominantly inorganic in nature, which could be fabricated via chemical vapor deposition (CVD). As shown in Fig. 4d, the memristor of MoS_2_ exhibits ultra-low variation and reliable memristive switching behaviors [86]. As shown in Fig. 4e, asymmetric heterostructure based on NbS_2_/NbOx was formed by natural oxidation after epitaxial growth on MoS_2_, resulting in a MoS_2_/NbS_2_/NbO_x_ structure [131]. The heterostructure-based memristor shows efficient tunneling conductivity and excellent memristive characteristics. Transition-metal phosphorus trichalcogenides are emerging 2D layered materials with formula of MPX_3_. Peng et al. developed a flexible memristor using CdPS_3_ nanosheets as the functional layer with the average thickness of 33 nm, demonstrating the application potential of CdPS_3_ in memristor (Fig. 4f) [14].

With great mechanical properties, electrical conductivity, high thermal conductivity and thermal stability, CNT is considered as typical 1D materials for future flexible memristors. These materials are generally classified as inorganic materials. As shown in Fig. 4g, single-walled carbon nanotubes (SWCNTs) were fabricated on ultra-thin flexible substrates [132], which exhibit superior synaptic properties. For wearable textile electronics, Wang et al. proposed a reconfigurable fiber memristor based on structure of Ag/MoS_2_/HfAlO_x_/CNT. The fiber memristor achieves non-volatile synaptic plasticity and volatile neuronal function, which could be used for ANN and SNN computing (Fig. 4h) [48]. By constructing 3D neural network based on 1D fiber memristor, the reconfigurable textile memristors exhibit great potential in future hybrid neuromorphic computing hardware. In addition, Van der Waals Integration technology could be used to construct 1D material-based memristor (e.g., CNT-Molecule-CNT memristor), as shown in Fig. 4i [89]. The length of the azobenzene molecule was approximately 2.6 nm, providing potential candidate for future size reduction of flexible electronics.

With unique quantum limiting effect and high specific surface area, zero-dimensional (0D) quantum dots showed great application potential in high-efficiency quantum conductive flexible memristors. 0D materials could be categorized as inorganic nanomaterials, owing to crystalline inorganic cores and dominant covalent/ionic bonding characteristics. As shown in Fig. 4j, biocompatible diffusion memristor based on nitrogen-doped graphene oxide quantum dots (N-GOQDs) was proposed to mimic biological synaptic functions [93]. The study demonstrated that N-GOQDs could serve as an insulating layer with a uniform thickness of ~ 130 nm. Core–shell quantum dots with quasi-type II band alignment of core–shell InP/ZnS quantum dots were successfully introduced to memristor [4], where photoexcited electrons are confined to the InP core while photoexcited holes are distributed in the ZnS shell (Fig. 4k). This structure, termed InP/ZnS switching layer (~ 20 nm thick), enables precise control of conductive filaments (CF) formation and dissolution under light irradiation. In addition, an innovative method for manufacturing all-inorganic perovskite quantum dots light-emitting memory (LEM) was proposed for flexible electronics, as shown in Fig. 4l [90]. The study demonstrated that CsPbBr_3_ quantum dots could act as core functional layer of memristor with thickness of approximately 800 nm.

Despite significant progress in the development of flexible materials, several key challenges still need to be addressed. The compatibility of low-temperature processes with CMOS technology, the nonlinearity of synaptic weight updates, and the reliability remains critical issues for large-scale flexible 3D integration. For 2D materials, stability and uniformity for large areas integration still need to be resolved. For 1D materials, achieving consistent and scalable fabrication processes is essential. For 0D quantum dots, the mechanism of quantum effect in memristor mechanism should be further studied for improving the operating voltage and uniformity of memristor.

## Structure of Flexible Memristor

**Fig. 5:**
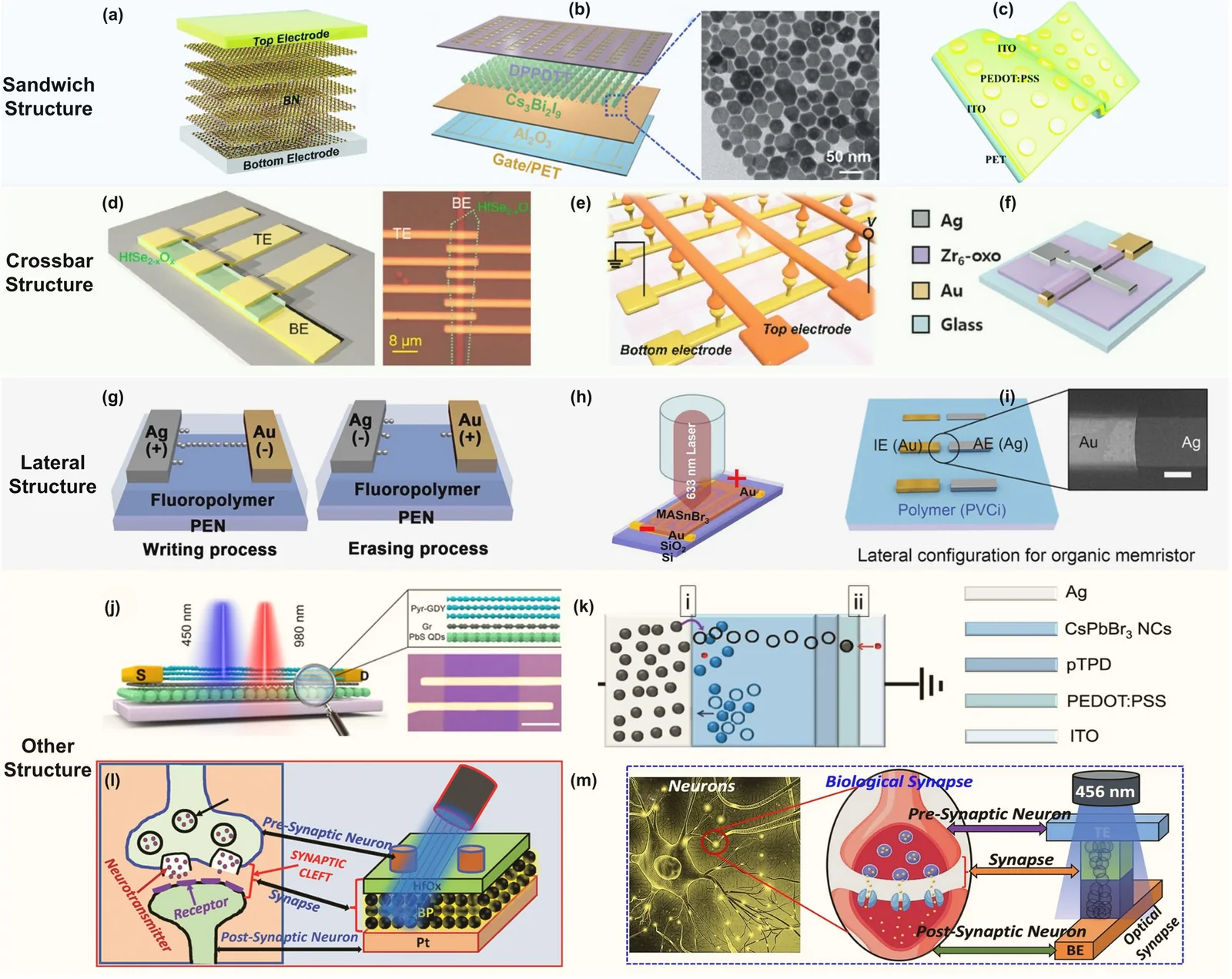
Common structure of the flexible memristor. Sandwich structure: **a** Sandwich structure of the ITO/BN/TaN memristor [92]; Copyright (2021) The Royal Society of Chemistry. **b** Schematic diagram of the Cs_3_Bi_2_I_9_-based optoelectronic synapses device, with a TEM image of Cs_3_Bi_2_I_9_ [97]; Copyright (2022) Wiley–VCH. **c** Schematic of transparent artificial synaptic device [137]. Copyright (2019) The Royal Society of Chemistry. Crossbar structure: **d** Schematic and optical microscope image of a typical HfSe_2−x_O_x_ memristor with crossbar structures [94]; Copyright (2020) American Chemical Society. **e** Schematic diagram of 3D crossbar synapses array [79]; Copyright (2020) American Chemical Society. **f** Schematic of single crossbar electrode configuration [106]. Copyright (2024) The Authors. Lateral structure: **g** Schematic of a fluoropolymer-based memristor with lateral structure [138]. Copyright (2021) The Authors. **h** Schematic of lateral-structured perovskite MASnBr3-based memristor [139]. Copyright (2019) The Authors. **i** Schematic of the lateral device structure for analyzing the conduction mechanism in organic memristors [22]. Copyright (2019) The Authors. Other structures: **j** Schematic of the optical synapse with floating gate (scale bar, 25μm) [140]; Copyright (2021) American Chemical Society. **k** Device structure consists of multi-type materials heterostructure, including PEDOT:PSS, poly-TPD and CsPbBr_3_ NCs [141]. Copyright (2022) The Authors. **l** Schematic of artificial optoelectronic synaptic device with heterostructure of HfO_x_ and BP [142]. Copyright (2023) The Authors. **m** Illustration of biological synapses and artificial optoelectronic synaptic devices [119]. Copyright (2024) The Authors

Sandwich structure-based memristor composed of metal/insulation layer/metal achieves resistive switching characteristics through design of interlayer interactions [6]. As shown in Fig. 5a, a low-power memristor with Excellent bipolar resistive switching characteristics was demonstrated by introducing BN nanosheets layer between top electrode (ITO) and bottom electrode [92]. Similar characteristic was demonstrated in inorganic/organic device based on sandwich structure of Cs_3_Bi_2_I_9_/PMMA/DPPDTT (Fig. 5b) [97]. As the organic device, organic artificial synaptic device demonstrated a super transparency and flexibility, because of the simple sandwich structure [137].

As a typical solution of 3D high-density integration, crossbar structure significantly improves the storage density and parallel computing capability of memristors. The primary distinctions between typical and vertical crossbar arrays are architecture and performance: typical arrays utilize 2D metal wiring with limited density, while vertical arrays employ 3D stacking with interlayer vias to achieve higher density, lower RC delay, and power reduction. However, vertical structures require advanced fabrication techniques (e.g., high-aspect-ratio via etching) and complex fabrication process compared to single-layer planar structure. 3D crossbar architectural evolution shows great potential in neuromorphic computing and high-density memory applications. As shown in Fig. 5d, unipolar memristor based on 2D HfSe_2-x_O_x_ was designed with switching ratio exceeded 10^6^, achieving logic and storage functions [94]. 3D crossbar memristor network has shown great potential in high-density integration. For example, Wang et al. proposed 3D Pt/HfAlO_x_/TaN memristor network through low temperature atomic layer deposition (Fig. 5e) [79]. The 3D flexible memristor network exhibits ultra-low power consumption of 4.28 aJ (lower than fJ level of biological synapses), high operation speed of 50 ns, and great fault-tolerant pattern recognition capability. As shown in Fig. 5f, single memristor with basic crossbar structure was developed using zirconium oxygen clusters (Zr₆O₄OH₄(OMc)₁₂) film as resistive switching layer, where the cluster network stiffness was regulated by thermal polymerization [117]. The random growth of conductive filaments (CFs) was inhibited in the memristor, forming a dispersed Ag cluster conductive pathway.

Above memristors are crossbar architecture, exhibiting reliable resistive switching characteristics. Another common configuration of memristor is the lateral structure [143, 144], characterized by coplanar alignment of electrodes with functional layer. With advantages of surface exposure, lateral structures are usually used to investigate conduction mechanisms to assistance analyzation of vertical structures. As shown in Fig. 5g, lateral memristor was specifically designed to explore growth process of conductive filaments [138]. Through field-emission scanning electron microscopy (FE-SEM), the active surfaces under different resistance states (LRS and HRS) were systematically measured, providing direct evidence of mechanism of memristors. As shown in Fig. 5i, the Ag conductive filaments in organic memristors were revealed by lateral structure [22]. This structural also design facilitates the fabrication of lateral lead-free perovskite-based memristor (Fig. 5h) [139], which exhibits remarkable mechanical robustness after 1000 bending cycles. Other common structures were summarized for novel applications. As shown in Fig. 5j, an optical synapse based on the vertical heterostructure with modulating gate was proposed, consisting of Pyrenyl-Graphdiyne (Pyr-GDY) /graphene/PbS quantum dots (PBS-QD) [140]. In addition, reconfigurable memristor based on halide perovskite nanocrystals was developed for switching between diffusion/volatile and drift/non-volatile modes (Fig. 5k) [141]. By selecting suitable perovskite nanocrystals and organic ligands, high performance switching between the two modes could be realized.

Recent studies have adopted the back-etching silicon technology to fabricate flexible memristors from rigid silicon. As shown in Fig. 5l, a novel optoelectronic synapse was developed by introducing the back-etching silicon technique, providing candidates for flexible neuromorphic computing [142]. Figure 5m presents another optoelectronic memristor under visible light, which utilizes a silicon back-etching process for bending [119]. The flexible device exhibits stable synaptic characteristics even when bent to a radius of 1 cm. These multifunctional features within single memristor make it suitable for applications in optoelectronic neuromorphic computing and artificial visual perception.

Different architectures exhibit distinct advantages and challenges, which were summarized in Table 2. The sandwich structure enables tunable properties, simple fabrication process and low power operation, which faces limitations of interfacial defects and scalability. The vertical crossbar offers high density and 3D integration advantages, which faces limitations of crosstalk, mechanical fragility and complex read–write circuits design. The lateral structure provides mechanisms and optoelectronic insights, which suffers from low integration density and high operation voltage. Other structures (e.g., back-etched Si) achieve CMOS compatibility at the expense of stress concentration. To balance performance with scalability and reliability, material innovations and advanced fabrication techniques should be studied to optimize device architectures. Moreover, distinct device architectures can be tailored for specific functionalities. Comprehensive emulation of synaptic behaviors was realized in a two-terminal Li-based device, presenting a promising solution for bio-inspired neuromorphic hardware [145]. In this work, sandwich structure was used for characterization and vertical crossbar structure was used to construct neuromorphic hardware system. By integrating an artificial neural network for image recognition tasks, unique synaptic properties of Li-ion-mediated artificial synapses were implemented successfully.

**Table 2:** The advantages and challenges of the different structures

Structure Type	Advantages	Challenges
Sandwich structure	Simple structure Low-power operation BEOL-compatible	Interfacial defects due to thermal mismatch Scalability limited by layer alignment precision
Crossbar structure	Ultrahigh density Energy-efficient parallel computing 3D integration potential	Crosstalk problem in high-density arrays Mechanical fragility Additional read–write circuits design
Lateral structure	Easy to analyze mechanism Large illuminated area for optoelectronic memristor	Low integration density Higher operating voltages
Others structure	Multifunction modulation Excellent CMOS compatibility	Failure caused by stress concentration High cost

## Performance of Flexible Memristor

### Method of Mechanical Deformation

**Fig. 6:**
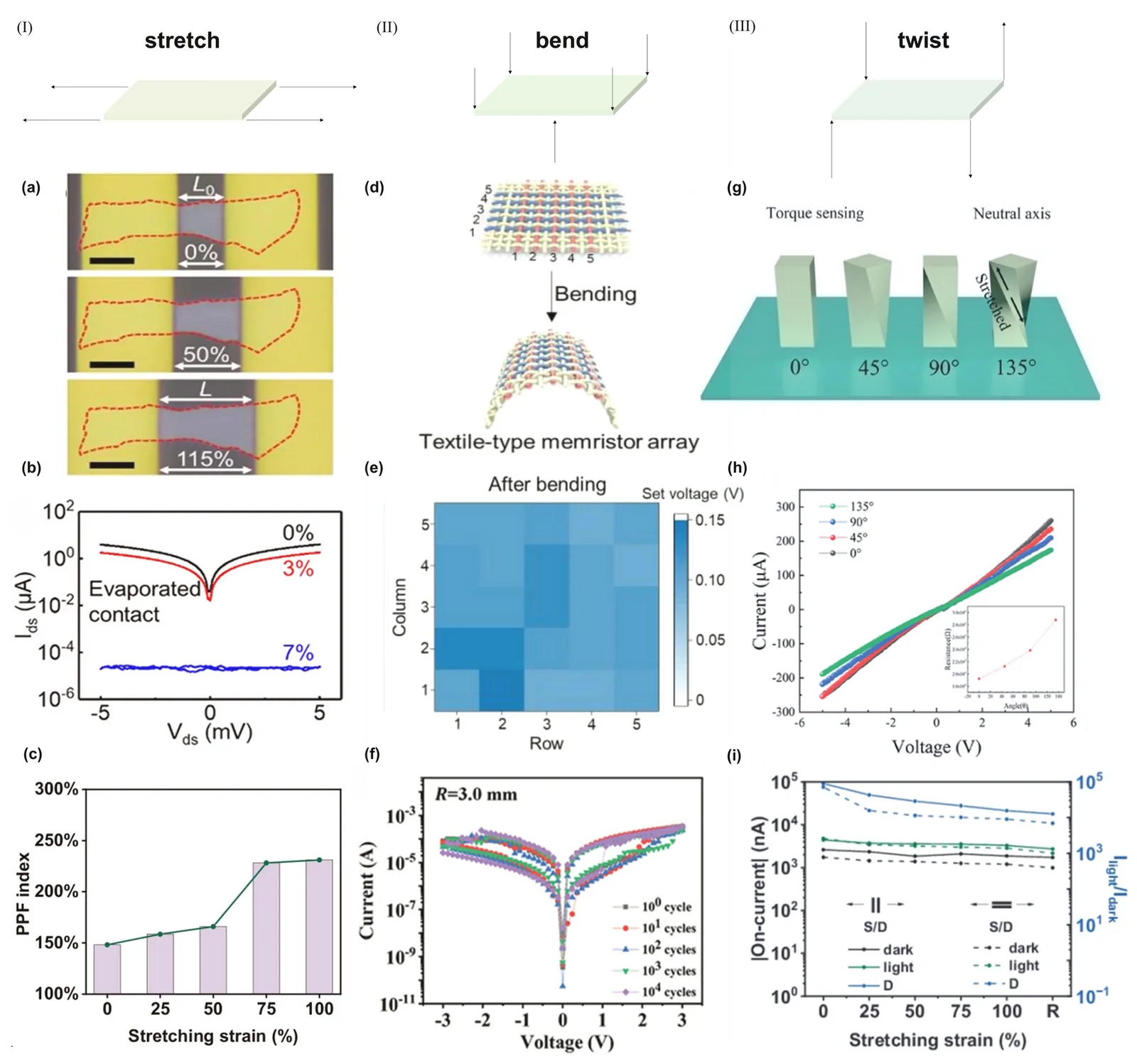
**(I)** Flexible device under stretching operation. **a** Schematic diagrams of functional layer with/without strain. With a weakly interacting Au − graphene interface, the Au electrodes can slide on the graphene surface during straining, releasing the applied strain [37]; Copyright (2022) American Chemical Society. **b** Log-scale I − V curve of device under different stretching operation, including 0%, 3%, and 7% [37]; Copyright (2022) American Chemical Society. **c** PPF index characteristic of device under different stretching strains [41]. Copyright (2024) The Authors. **(II)** The flexible device under bending operation: **d** Schematic diagram of the textile memristor under bending operation [98]; Copyright (2023) Wiley–VCH. **e** Statistical results of set voltage of 25 memristor units before and after 100 bending cycles [98]. Copyright (2023) Wiley–VCH. **f** I − V curves of memristor after repeated bending cycles [24]. Copyright (2023) Wiley–VCH. **(III)** Flexible device under twisting operation: **g** Twisting operations under different angle [40]; Copyright (2024) The Authors. **h** Current response of device under different twisting angles [40]; Copyright (2024) The Authors. **i** Strain-tolerance characteristics of device, which involves stretching first and twisting last [41]. Copyright (2024) The Authors

This section mainly introduces the types of mechanical deformation, the parameters of mechanical deformation, the influence of mechanical deformation on device properties, and the simulation methods of mechanical deformation. Mechanical deformation can be categorized into three types by force directions applied to the device, including bending, stretching, and twisting operations. Stretching involves applying two forces of equal magnitude but opposite directions to the ends of the device, where forces act outward, as shown in Fig. 6a [37]. The resultant effect on device is an extension in the direction of the applied force, accompanied by slight contraction in the vertical direction (Fig. 6c) [41]. Moreover, stretching may lead to structural misalignment of functional layer, and cause direct damage when the stretching operation exceeds the endurance limit (Fig. 6b) [37] [146]. Conversely, when the two forces act inward, reverse stretching effect of compression could be induced, and even lead to a certain degree of bending for soft materials. Bending operation occurs when two forces of equal magnitude and downward directions are applied to the ends of the device, along with an upward force on the plane perpendicular to the device (Fig. 6d). This causes the device to deform into a bent shape, with the surface and base experiencing tensile and compressive forces, respectively. The surface of the device is more prone to damage under bending states, which may affect electrical performance of device (Fig. 6e, f) [24, 38, 98, 147]. Twisting operation occurs when applying forces in the same direction diagonally to the device, where two diagonal forces act in opposite directions. The force distribution on the surface of a twisted device is more complex, involving roughly stretching and certain amount of torque (Fig. 6g–i) [40, 41]. This type of deformation generally occurs linear devices. Due to different features of different mechanical deformation, few reported flexible memristors have conducted all types of mechanical deformation. Therefore, different structures and materials need to be adopted for various wearable application scenarios.

### Parameters of Mechanical Deformation

**Fig. 7:**
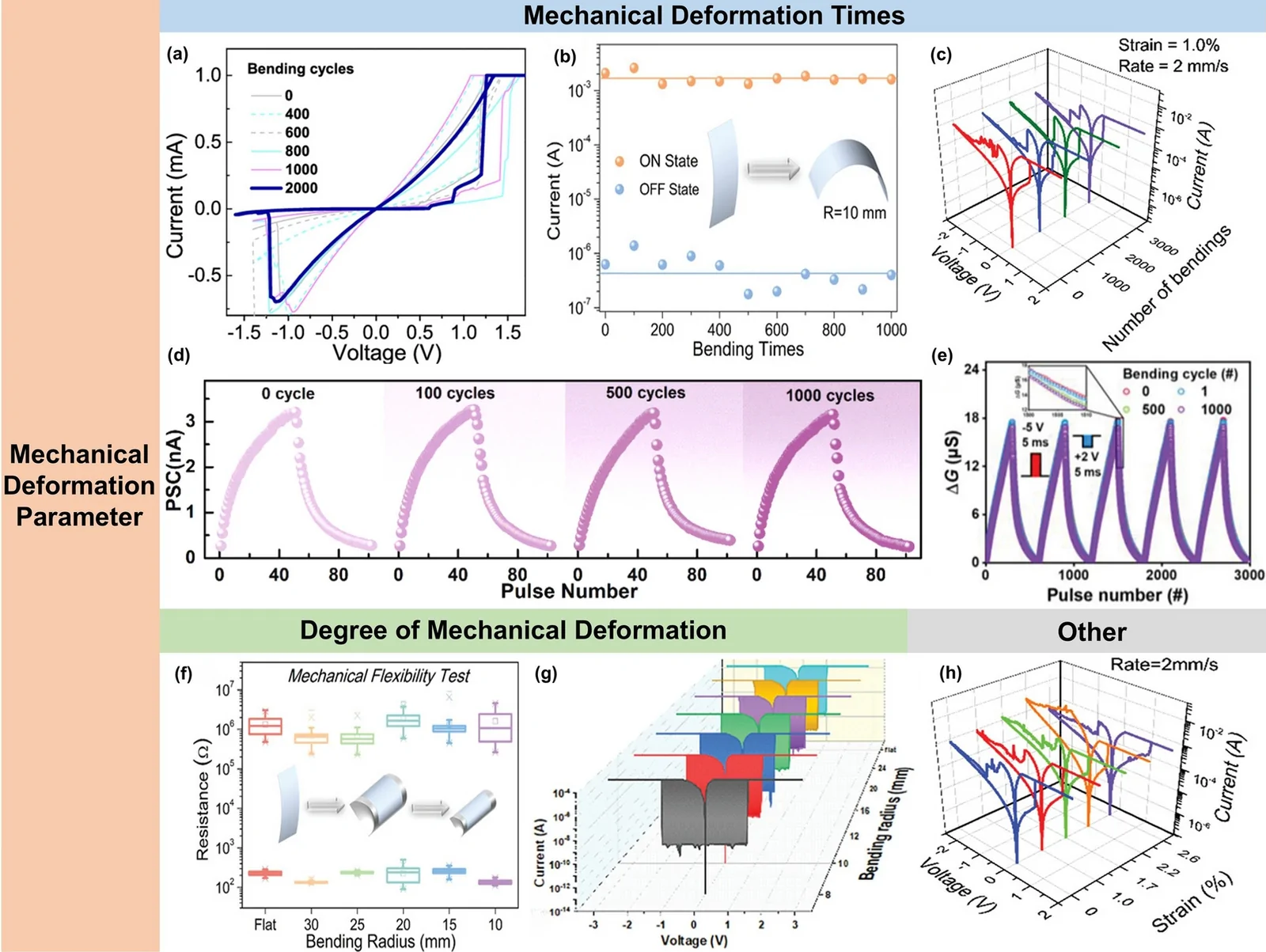
Variable bending parameters. **a** Bending cycles is used to describe the number of deformations of the device under specified mechanical deformation [131]. Copyright (2020) American Chemical Society. **b** ON/OFF current of device under different bending times [148]. Copyright (2024) Wiley–VCH. **c** Resistive switching curve of device under different bending times [100]. Copyright (2021) Wiley–VCH. **d** LTP/LTD curve of synaptic memristor under different bending cycles [101]. Copyright (2021) Elsevier Ltd. **e** Conductance change under different pulse number and bending cycles [99]. Copyright (2024) Wiley–VCH. **f** Bending radius is generally used to verify the application environment under different degree of mechanical deformation [148]. Copyright (2021) Wiley–VCH. **g** I-V curves of memristor under different bending radius [102]. Copyright (2024) Wiley–VCH. **h** I-V curves of memristor under different strain [100]. Copyright (2021) Wiley–VCH

Mechanical deformation parameters exhibit variations among bending, stretching, and twisting operations. This section illustrates common variables in mechanical deformation with bending as an example. The first parameter of evaluating flexible electronics is bending cycles, which reflects the endurance characteristic of device under bending operations, as shown in Fig. 7a–e. In bending operation, the degree of mechanical deformation is indicated by the curvature at the bending point, namely as bending radius he bending radius (1–25 mm). Smaller bending radius signifies greater degree of mechanical deformation and more substantial impact on the device (Fig. 7f, g) [102, 148]. The performance of the device under a certain bending radius can be used to verify the reliability and functionality of the flexible memristor [13, 24, 72, 77, 98, 101, 148–150]. The smallest bending radius of flexible memristor represents the limits of flexible device performance during bending operations. Additionally, the statistical electrical performance of device with the bending radius can be plotted to investigate the physical causes resulting from mechanical deformation, providing guidelines for performance improvement [151, 152]. In stretching operations, the degree of mechanical deformation is represented by the tensile percentage [37, 153, 154]. The definition of tensile percentage is difference between the stretched length and the original length divided by the original length of the device. In twisting operation, the degree of mechanical deformation is defined by the angle compared to the reference plane of the original device, generally ranging from 0 to 180 degrees. The dynamic test is repeated mechanical deformations refers to the number of bending, stretching or twisting at a certain degree of mechanical deformation [24, 100, 101, 131, 148]. It is generally considered that one cycle mechanical deformation is completed when the device is bent and then reset, which is the basic of dynamic measurement under repeated cycles [24, 38, 99–101, 148, 149]. The number of mechanical deformations can be utilized to analyze the fatigue process of flexible device [11, 155, 156]. The remaining parameters are directly plotted with stress or strain percentage (Fig. 7h), which could be used in complex mechanical deformation scenarios [41, 100].

Figure 7a demonstrates the performance of NbS_2_ memristors after thousands of bending cycles with a bending radius of 10 mm [131]. The results show that the devices maintain excellent I–V characteristics after 2000 bending cycles, indicating superior mechanical durability. Figure 7b presents the evolution of ON/OFF current under different numbers of bending cycles (10 mm radius), demonstrating the flexible reliability of memristor after 1000 bending cycles [148]. Figure 7c displays I-V characteristics after thousands of bending cycles at fixed strain (1% strain, 2 mm s^−1^), further verifying the fatigue resistance [100]. The repeatability of conductance modulation under different bending cycles determines the application potential of device in flexible neuromorphic electronics. Figure 7d presents long-term potentiation/depression (LTP/LTD) characteristics of flexible optoelectronic artificial heterosynapse with functional layer of 14 nm MoSSe [101], proving excellent mechanical fatigue. Similar characteristics of LTP/LTD under 3000 repeated bending cycles was demonstrated in Fig. 7e [99]. Figure 7h employs generalized strain percentage parameter (commonly used in simulations), showing classical I-V curves to verify whether electrical performance degrades under different strain conditions [100].

### Improvement of the Performance

**Fig. 8:**
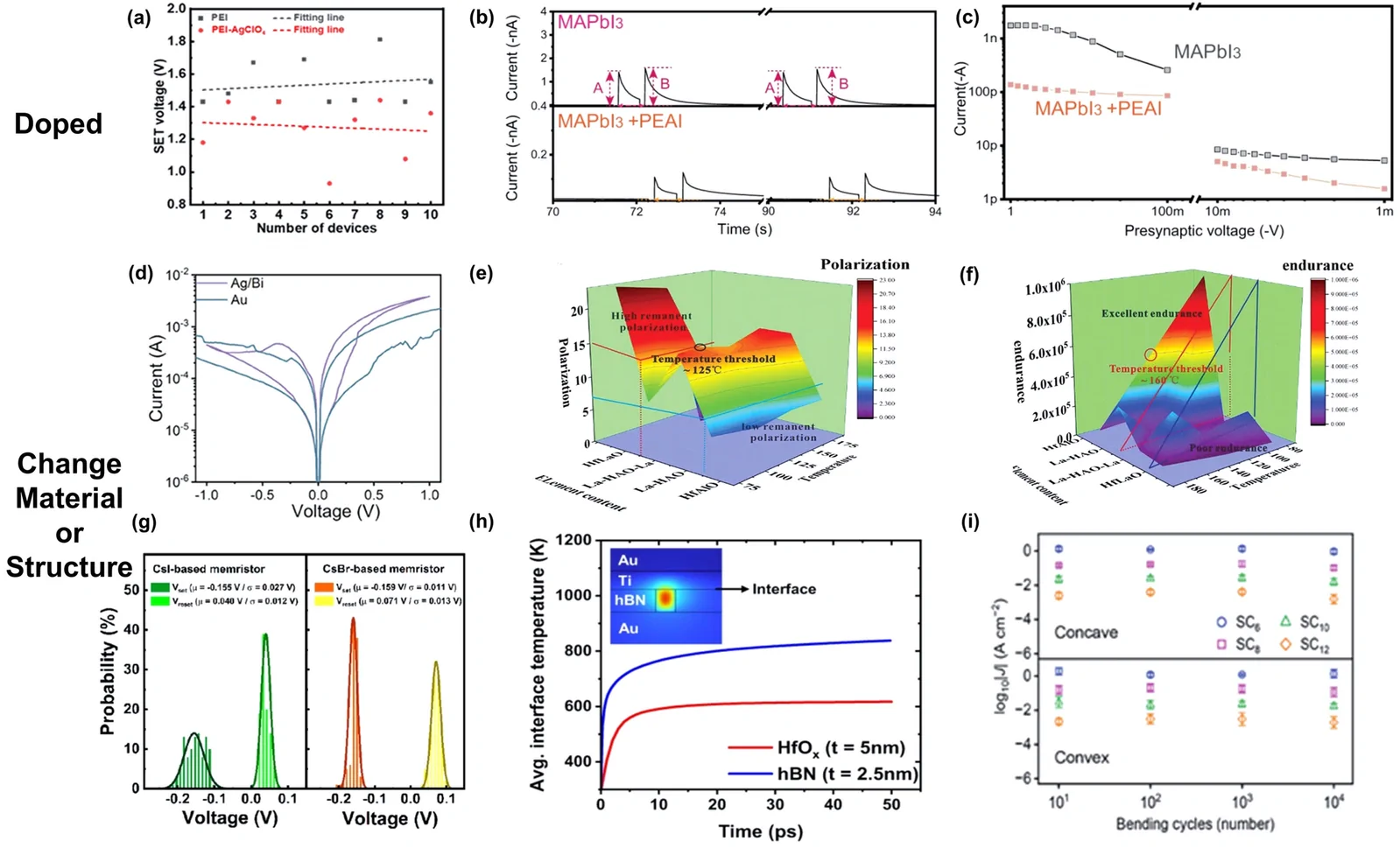
Improvement of memristor performance, including materials doping and structure design. **a** Comparison of the SET voltage for 10 devices with/without AgClO_4_ doping operation [106]. Copyright (2022) American Chemical Society. **b** EPSC response of perovskite artificial synapse with/without PEAI passivation [104]. Copyright (2022) The Authors. **c** Different current vs pre-synaptic voltage of device with/without PEAI [104]. Copyright (2022) The Authors. **d** I − V curves of memristors with different electrode, including Ag/Bi and Au [108]. Copyright (2025) Wiley–VCH. **e** Remanent polarization of memristor under different element content and temperature [105]. Copyright (2024) The Authors. **f** Endurance characteristic of memristor affected by element content [105]. Copyright (2024) The Authors. **g** Set and reset voltage distributions of devices of CSI and CsBr [107]. Copyright (2022) American Chemical Society. **h** COMSOL-simulated transient interface temperature of memristor with functional layer of hBN and HfO_x_ [55]. Copyright (2024) The Authors. **i** Current density of devices of SC_6_, SC_8_, SC_10_, and SC_12_ under different bending cycles. Error bars indicate standard deviation [95]. Copyright (2024) Wiley–VCH

The methods to improve device performance can be summarized as materials doping and structure design. As an effective method to improve the performance of flexible memristors, incorporating conductive particles into functional material could improve the electrical characteristics. For example, AgClO_4_ doping could reduce operation voltage, increase switching speed and reduce dispersion of the SET voltage (Fig. 8a) [106]. By introducing specific dopants into the memristor, the uniformity and stability of the memristor can be significantly improved. As a common dopant, PEAI (phenyl ethyl ammonium iodide) is widely used in perovskite-based memristors. Doping of PEAI can improve the switching ratio and durability of the device, enabling it to achieve efficient resistance switching under low voltage (Fig. 8b, c) [104]. In addition to doping materials, structures design is also an effective way to improve the performance of flexible memristors. By changing the electrode material or introducing new structure, the performance of the memristor can be significantly improved. The current–voltage (I-V) characteristic curves under different electrode materials (Ag/Bi and Au) show that the introduction of Ag/Bi electrode significantly improves the electrical conductivity and switching characteristics of the device (Fig. 8d) [108]. By changing the distribution of the material, the conduction mode could be modulated to improve the performance of device. For example, unique interfacial memristor in all-inorganic flexible memristor was proposed with solid electrolyte [157]. To overcome the shortcomings of single-layer WO_x_, the HfO_x_ ion diffusion layer was introduced to improve the switching characteristics of original device [158]. It is also feasible to change the material structure or composition to improve device performance. For example, the alkanethiol molecules with different chain lengths (SC_6_, SC_8_, SC_10_, SC_12_) were used to form SAMs (Fig. 8i) [95]. Under different bending times, materials with different chain lengths have different current densities. As shown in Fig. 8e, f, La doping in functional layer enhances the properties of ferroelectric memristor [105]. Moreover, different substrate can also be used to adjust the performance, such as CsI and CsBr. As shown in Fig. 8g, voltage response of the memristor is different under different substrates [107]. The results of the interface temperature of HfO_x_ and h-BN under ultrafast pulse reveals the thermal stability and operation speed could be directly modulated by different types of functional layer (Fig. 8h) [55]. The device structure design can also improve the performance of the memristor. After introducing PMMA to GeSbTe, the device shows excellent multi-level storage capability [96]. By adding insertion layer of ZrO_2_, the ferroelectricity of memristor was enhanced [159]. In addition to above approaches, back-etching method was used to direct fabricate flexible device based on rigid silicon. The flexible silicon-based device exhibits similar performance compared with that on rigid silicon substrate [160]. By materials doping, structure design and novel fabrication processes, the performance of flexible device could be enhanced.

### Bending Simulation

**Fig. 9:**
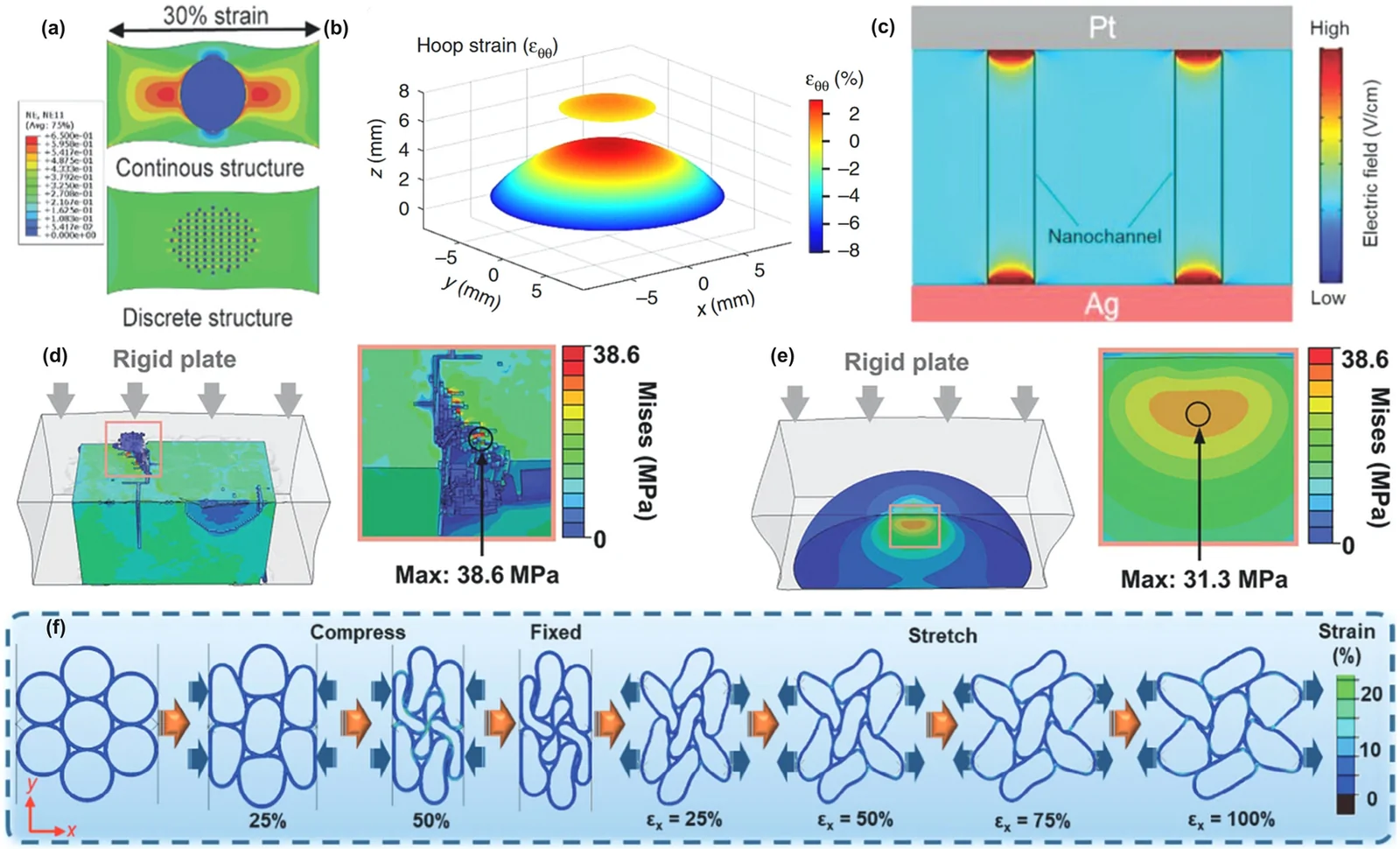
**a** Finite element analysis (FEA) simulations of strain distributions in continuous and discrete structures under 30% tensile strain of 30%, revealing that the discrete structure exhibits highly reduced strain concentration [116]. Copyright (2021) Wiley–VCH. **b** Radial and hoop strain patterns of films [43]. Copyright (2017) The Authors. **c** Electric field simulation of memristor with nanochannels, revealing localized field enhancement at nanochannels [98]. Copyright (2023) Wiley–VCH. **d** Stress distribution in square-shaped ceramic composites under rigid plate loading [161]. Copyright (2024) The Authors. **e** Stress distribution in droplet-shaped ceramic composites under rigid plate compression [161]. Copyright (2024) The Authors. **f** FEA simulations of pre-stretched semiconducting aerogel films during release and stretching at 100% strain [146]. Copyright (2024) Wiley–VCH

The simulation technology of flexible memristor plays a crucial role in guidelines of device design. Finite element simulation is a typical simulation method for analyzing stress distribution. The steps include drawing the model, allocating the materials of each part, finite mesh division, output parameter setting. Generally, load and boundary conditions are applied to determine which mechanical deformation method is used. The performance changes of the flexible memristor under different mechanical deformation, the associated stress distribution and crack growth process could be analyzed for performance optimization. As shown in Fig. 9a, the stress distribution of continuous and discrete structures under 30% strain were analyzed [116]. The simulation results show that the continuous structure produced obvious stress concentration under high strain, while the discrete structure effectively dispersed the stress and reduced the risk of structure damage. This analyzation of stress distribution is important for understanding the mechanical stability of flexible memristors. As shown in Fig. 9b, flexible optoelectronic devices exhibited significant advantages in mechanical compatibility, which have minimal interference to the eye model by comparing the strain distribution of devices attached to the eye model [43]. The flexible optoelectronic devices exhibited advantages in reducing mechanical damage and improved biocompatibility compared with traditional ring device.

As shown in Fig. 9c, the electric field simulation result demonstrates that the nanochannel promotes ion migration under the action of electric field, resulting in the formation of ordered conductive filaments and reliable resistance switching behavior [98]. The simulation result provides effective guideline for improve device performance by introducing nanochannel. As shown in Fig. 9d, e, the stress distributions of the droplet shape under rigid plate and compression of rigid plate were used to analyze the maximum stress value [161]. The smaller stress value means better durability, which is important for the reliability of flexible memristors in practical applications. The deformation process of semiconductor aerogel film under different strain conditions are shown in Fig. 9f [146]. These simulation results verify the mechanical stability of the discrete structure under high strain, revealing adaptability under different deformation modes. The result has important guiding significance for designing stable flexible memristor in complex mechanical environment.

## Application of Flexible Memristor

**Fig. 10:**
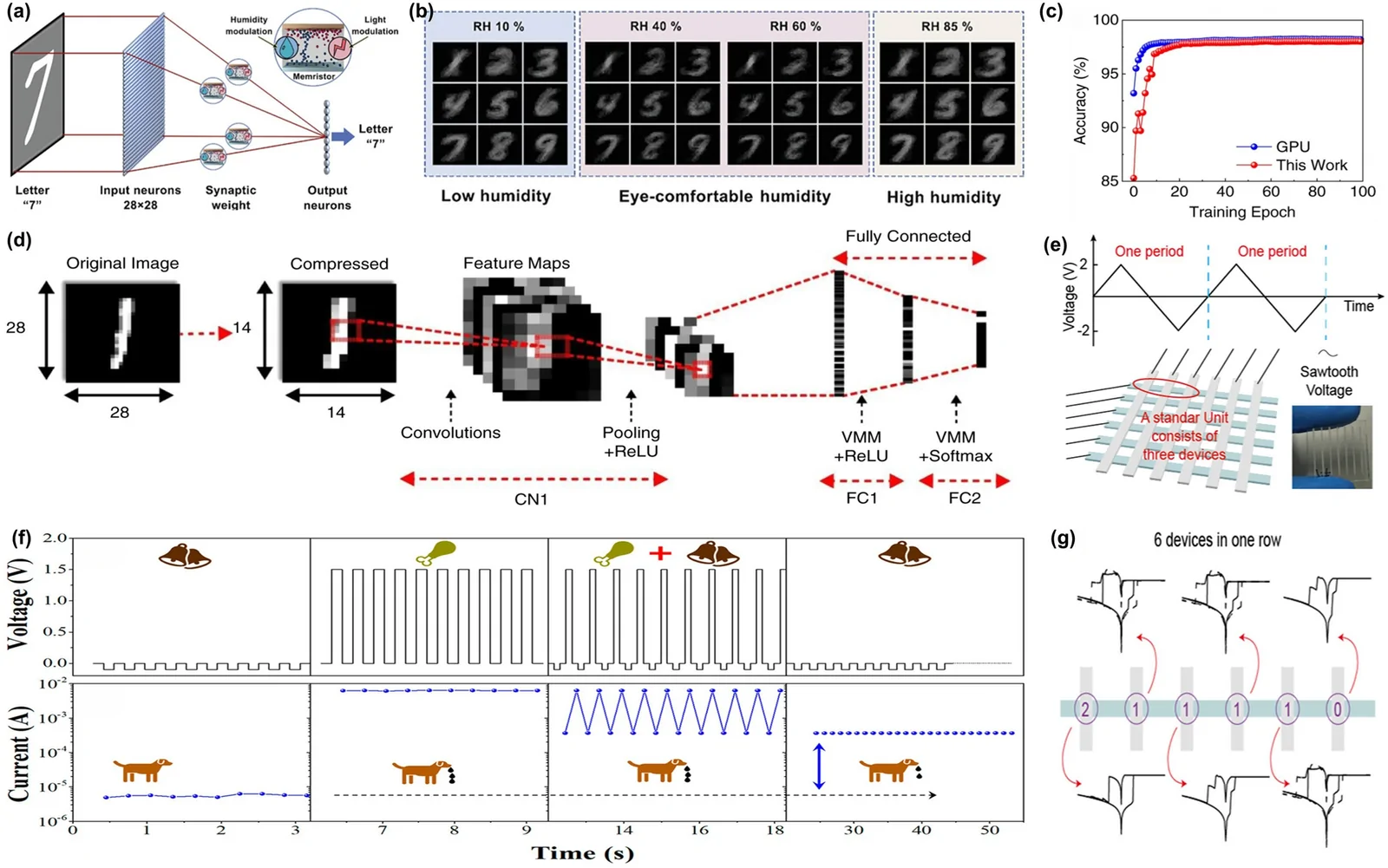
Application of flexible memristor for synaptic plasticity. **a** Visual depiction of in-sensor computing using optoelectronic memristor for weight adjustment [88]. Copyright (2021) Wiley–VCH. **b** Output results of memristor under varying humidity conditions [88]. Copyright (2021) Wiley–VCH. **c** Accuracy of memristor-based 3-layer CNN versus GPU processing over 100 training cycles [87]. Copyright (2022) The Authors. **d** CNN architecture for handwritten digit classification [87]. Copyright (2022) The Authors. **e** Schematic of 6 × 6 crossbar array using three row devices as functional units [91]. Copyright (2020) American Chemical Society. **f** Implement of classical conditioned reflex behavior in Pavlov dogs, where different voltage sequences represent different signals, including bell, food, and bell plus food [31]. Copyright (2024) American Chemical Society. **g** Analyzation of sequential resistive state transitions of different memristors by Markov chain modeling [91]. Copyright (2020) American Chemical Society

With advantages of resistive switching characteristics, ultra-low power consumption and low cost, flexible memristors have emerged as promising electronic components for neuromorphic computing. These attributes enabled the development of bio-inspired synaptic devices and flexible artificial neural networks. Device conductance could be modulated under different pulse stimuli, similar as the process of weights update in bio-synapse. Inspired by human visual system, optoelectronic memristors exhibit great advantages in in-sensor computing. As shown in Fig. 10a, b, MXene-ZnO heterostructure-based memristor can be utilized for multimodal in-sensor computing by modulating oxygen vacancy filaments via light under different humidity, which could realize retinal adaptation functions [88]. In this work, Kelvin probe force microscopy (KPFM) and electron energy-loss spectroscopy (EELS) confirmed photon/proton-coupled switching mechanisms, laying the foundation for integrated sensing and computation. As a typical neuromorphic computing task, image recognition was achieved by wafer-scale MoS₂ memristor arrays, as shown in Fig. 10c, d [87]. The memristor array achieved high uniformity (< 8% device variation) and reached 98.02% accuracy in MNIST recognition tasks. Based on the random modulation process of conductive filaments, true random number generation and probabilistic computing were realized in memristor array (Fig. 10e, g) [91]. Such stochasticity aligns with biological neuronal spiking, offering pathways for brain-inspired probabilistic architectures. As a classic conditioned reflex experiment, the saliva secretion experiment of Pavlov dogs was validated by memristor. as shown in Fig. 10f. Solution-processed MoS₂-based memristor (functional layer of 25 nm) exhibit excellent synaptic plasticity, paving the way for high-order learning behaviors [31].

**Fig. 11:**
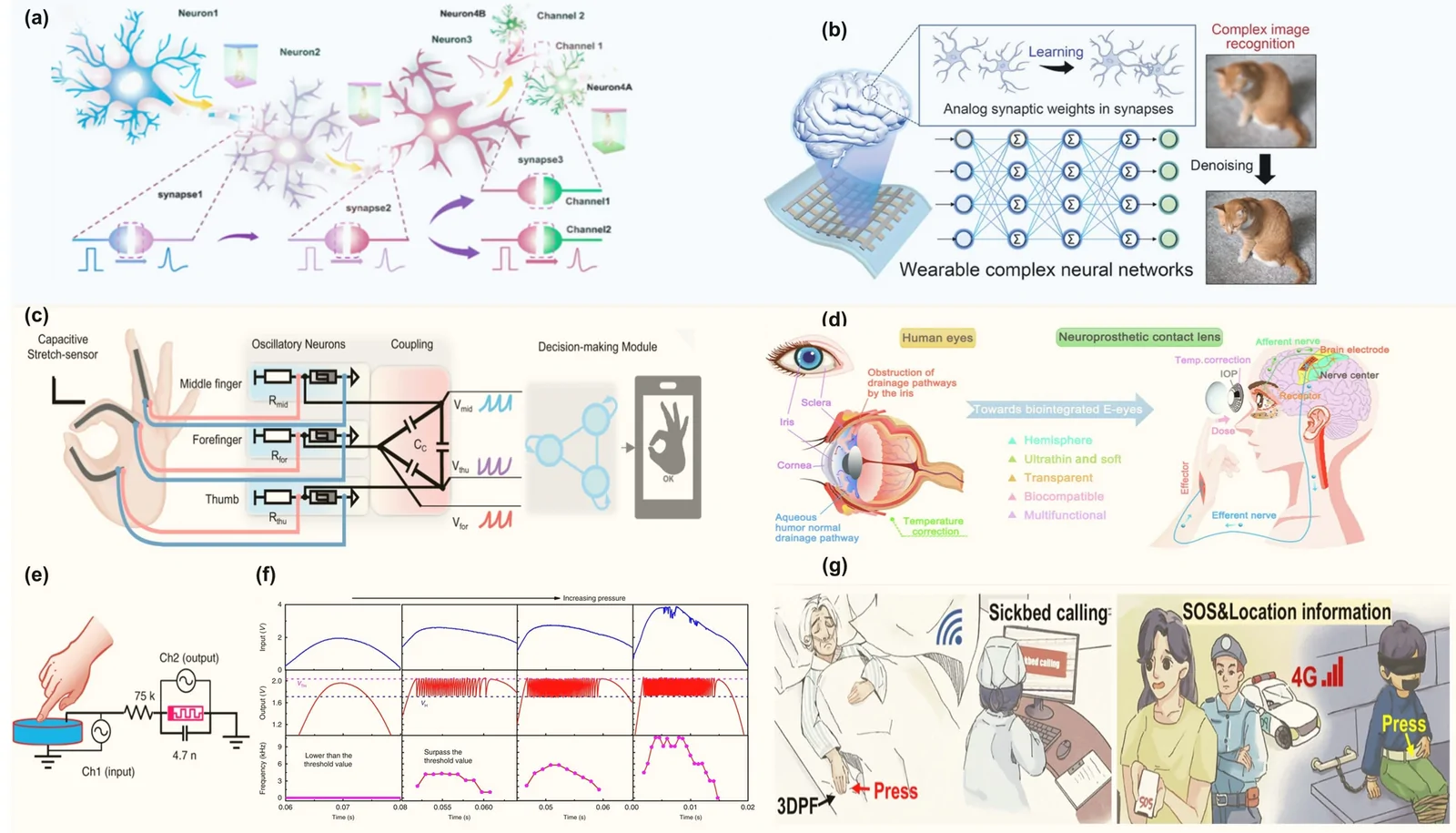
Application of memristor for neuromorphic computing. **a** Diagram of biological neural network for information transmission through multiple pathways [79]. Copyright (2020) American Chemical Society. **b** Wearable visual neural networks for image denoising processing [109]. Copyright (2023) The Authors. **c** Neuromorphic sensing system designed for gesture detection, where the signals are processed for classification task [81]. Copyright (2024) The Authors. **d** Inspired by the human eye's anatomy, where the cornea, iris, and sclera undergo significant deformation under high intraocular pressure, neuromorphic artificial circuit based on neuroprosthetic lens was constructed [46]. Copyright (2024) The Authors. **e** Artificial nerve consisting of memristor and tactile sensor. The output voltage of sensor acts as the input signal of memristor [27]. Copyright (2020) The Authors. **f** Response curve of artificial nerve under different stimuli [27]. Copyright (2020) The Authors. **g** Smart healthcare application scenarios. Memristor-based smart system used for hospital bed alerts and wearable belt for tracking missing children [42]. Copyright (2024) The Authors

In the aspect of device structure design, three-dimensional integration and multimodal fusion have become critical design strategies. Wang et al. developed a flexible 3D HfAlO_x_-based memristor network with ultra-low energy consumption (4.28 aJ per synaptic event) and 50 ns response speed (Fig. 11a) [79]. The 3D neural network based on flexible memristor exhibits short-term plasticity and LTP/LTD for noise-tolerant image recognition. As shown in Fig. 11b, engineered cluster-structured metallic filaments in polymer matrices were designed for 64 states storage and image denoising processing in wearable neuromorphic systems [109]. High-order sensory processing nanocircuit based on coupled VO_2_ (~ 20 nm) oscillators and artificial neuron was designed for sensory preprocessing in continuous-time dynamic systems (Fig. 11c) [81]. The system encoded information in phase differences and included a decision module for special post-processing, demonstrating advantages in tactile and gesture recognition tasks with fewer device and lower energy. This research indicated the potential of flexible memristors in high-performance sensory systems. Flexible memristors also exhibit advantages in smart healthcare. Liu et al. proposed a neuroprosthetic contact lens sensor system for real-time monitoring and feedback of intraocular pressure (Fig. 11d) [46]. The system integrated a memristor and temperature sensor, achieving high sensitivity and accuracy through temperature compensation. This study highlighted the potential of flexible and biocompatible materials in medical monitoring applications. By emulating neuron functions, Mott memristor based on NbO_x_ (~ 25 nm) could be integrated into artificial spiking afferent nerve for neurorobotics (Fig. 11e, f) [27]. The system converted analog input signals into spike frequencies, mimicking the function of biological neurons. By using piezoelectric devices as tactile sensors, the system successfully captured pressure signals and converted pressure intensity into corresponding spike frequency, demonstrating the potential of flexible memristors in neural robotics and tactile sensing applications. In addition, flexible memristors could be fabricated as fiber shape for smart electronics [162]. 3D PVDF piezoelectric nanoyarn fabric strain sensor shows excellent breathability and ultrahigh strength, demonstrating the potential of flexible materials in wearable electronics (Fig. 11g) [42]. The PVDF nanofibers-based sensor was woven into functional yarns with different hygroscopic properties and then into a 3D fabric structure, exhibiting high sensitivity and fast response speed even under sweaty conditions.

For wearable neuromorphic computing integration, flexible neuromorphic computing system based on universal fundamental circuit units and essential peripheral circuitry was developed [163]. Flexible memristors can enable real-time adaptation and advanced responses in unstructured environments for future autonomous driving scenarios. Additionally, flexible memristors exhibit promise in brain-computer interfaces (BCIs). Memristor-based neuromorphic and adaptive BCI decoder were designed for achieving high precision, rapid response, and ultra-low power consumption brain signal decoding [164]. Furthermore, flexible memristors facilitate high-precision neuromorphic computing. Researchers demonstrated a graphene-based non-volatile resistive memory device capable of 16 distinct conductance states [165], enabling on-chip k-means clustering. Compared to uniform quantization, k-means clustering offers significant advantages in quantizing artificial neural networks (ANNs). Differential neuromorphic computing was implemented by memristor with the intrinsic multistate behavior of extracting features from unstructured data [166], thereby improving system adaptability in dynamic environments. In addition, advanced characterization techniques have been introduced to achieve neuromorphic computing functions, such as conductive atomic force microscopy (CAFM). Threshold response, relaxation, sensitization, and synaptic plasticity was realized in memristors based on CAFM [167]. This advancement holds significant implications for the development of high-precision neuromorphic computing and wearable/implantable biosensors in the field of semiconductor devices. Advanced fabrication process of low temperature promotes the development of flexible neuromorphic computing electronics. Wafer-level array integration based on CMOS-compatible process offers a promising solution for wearable neuromorphic computing applications [168]. In additional, new principle device was introduced to achieve neuron function for SNN computing. Antiferroelectric materials acting as functional layer of memristor could emulate neuronal behavior in single device, offering a promising approach for developing efficient neuromorphic hardware [169]. Spike-feature-driven sensorimotor neural circuit was developed for bio-inspired selective communication, thereby advancing the development of higher-order robotic systems [170].

Beyond the above enhancements in advanced biological synaptic systems, memristors play an indispensable role in in-memory multimodal computing, aiming to improve the processing of diverse sensor data types while reducing data transmission bottlenecks inherent in traditional computing architectures. Flexible memristor-based cross-modal spiking sensory neuron (CSSN) was proposed to process multimodal signals and provide tactile feedback for in-sensor computing [80]. By utilizing memristors to encode CSSN signals, in-memory multimodal perception for human–machine interaction was demonstrated. Similarly, a memristor-circuit-based multimodal neuromorphic sensory processing system was designed with low cost [171]. Furthermore, in-memory multimodal sensing technology can be applied to multifunctional image processing. Novel fully hardware-implemented vision system with multifunctional image processing capabilities was proposed for image preprocessing [172]. The system completely mimics preprocessing and processing functions of retinal cells and visual cortex. The emerging multimodal system features operational modes of OORAM and EERAM. The former enables in-sensor data optimization and convolutional operations, while the latter accomplishes in-memory image recognition. The emerging visual memristor presents a feasible approach for the development of future machine vision systems.

Current research still faces challenges such as material interface regulation, device uniformity improvement, and multi-scale integration. Future development of flexible memristors focus on developing functional materials with both mechanical flexibility and environmental stability, and developing cross-scale device-circuit co-design method [173]. The deep integration of flexible memristors with flexible sensors is expected to promote the development of adaptive neuromorphic systems to a higher level of intelligence [174, 175]. In the future, the application of flexible memristors in sensors will be biased toward robots and wearable electronics [47, 169, 176–179], providing hardware support for the development of bionic robots and wearable electronic devices.

## Challenge and Outlook

Although flexible memristors have an irreplaceable role and great development prospect in artificial synapse and neuromorphic computing, they still facing many challenges. On the material side, achieving large-scale fabrication and maintaining uniformity remains difficult, especially for low-dimensional materials (e.g., 2D, 1D, and 0D). The inconsistent synthesis methods and interface defects should be addressed for uniform resistive switching layer. For 3D bulk materials, compatibility with low temperature CMOS processes, easy to break, and synaptic weight nonlinearity limit the application potential of bulk materials in flexible neuromorphic systems. On the structure side, under repeated mechanical deformation (bending, stretching, twisting), stress concentration can lead to interface delamination or fracture of conductive filaments and damage performance. Recent studies indicate that low-dimensional materials exhibit exceptional resilience to mechanical deformation in flexible memristor applications, owing to unique structural and mechanical properties. For 2D materials of MoS₂, h-BN, and MXenes, atomic-scale thickness and weak van der Waals interlayer interactions enable intrinsic flexibility and strain redistribution through layer sliding, effectively mitigating crack propagation. Similarly, 1D carbon nanotubes (CNTs) leverage high tensile strength and axial flexibility, with nanotube networks uniformly distributing strain to prevent localized failure. In contrast, 3D bulk materials (e.g., HfO₂, TiO₂) suffer from interfacial delamination due to rigid lattice structures. 0D quantum dots face challenges of nanoparticle aggregation under strain. Therefore, 2D layered materials and 1D CNTs are prioritized for deformable electronics (e.g., wearables), where mechanical compliance and defect-tolerant architectures are critical for long-term reliability. In addition, achieving a balance between high-density integration, multifunction and mechanical flexibility requires innovative design. On the function side, device variability, high power consumption, and insufficient environmental stability (such as humidity sensitivity) further hinder their reliability in wearable applications.

In the future, research efforts should focus on enhancing material properties by exploring diverse material compositions and structural designs to address critical challenges such as non-uniform stress distribution and inferior electrical performance [37, 105, 116, 117]. Furthermore, advanced fabrication techniques must be systematically investigated to facilitate the scalable manufacturing of flexible memristors and accelerate industrial adoption [38, 52]. A promising direction lies in expanding the applications of flexible memristors, particularly in neuromorphic computing, intelligent robotics, and sensor systems. Owing to inherent operational similarity to human brain, memristors have demonstrated significant potential in neuromorphic computing. It is imperative to evaluate the performance gap between rigid memristors and flexible counterparts, as well as to explore whether flexible memristors can enable unique functionalities unattainable with rigid devices in specific application scenarios.

The development of flexible memristor technology holds significant promise for advancing neuromorphic computing, which embodies a paradigm shift toward energy-efficient, brain-inspired in-memory computing architecture. This transformative approach leverages key innovations in memristor, 3D integration, and spiking neural networks, enabling diverse applications from edge computing to brain-machine interfaces. As emerging technologies of quantum-inspired neuromorphic computing and biohybrid systems expand the application potential in artificial intelligence, the synergy of novel materials, scalable fabrication methods, and biologically plausible algorithms is poised to bridge artificial and biological intelligence. Notably, progress in flexible electronics may soon yield mass-producible, high-performance wearable memristors, which would not only accelerate neuromorphic computing advancements but also create profound impacts on everyday technologies.
